# The Cytotoxic Necrotizing Factor 1 from *E. Coli*: A Janus Toxin Playing with Cancer Regulators 

**DOI:** 10.3390/toxins5081462

**Published:** 2013-08-14

**Authors:** Alessia Fabbri, Sara Travaglione, Giulia Ballan, Stefano Loizzo, Carla Fiorentini

**Affiliations:** Department of Therapeutic Research and Medicines Evaluation, Superior Health Institute, viale Regina Elena 299, 00161 Rome, Italy; E-Mails: alessia.fabbri@iss.it (A.F.); sara.travaglione@iss.it (S.T.); giukkab@gmail.com (G.B.); stefano.loizzo@iss.it (S.L.)

**Keywords:** CNF1, cancer, inflammation, Rho GTPases

## Abstract

Certain strains of *Escherichia coli* have been indicated as a risk factor for colon cancer. *E. coli* is a normal inhabitant of the human intestine that becomes pathogenic, especially in extraintestinal sites, following the acquisition of virulence factors, including the protein toxin CNF1. This Rho GTPases-activating toxin induces dysfunctions in transformed epithelial cells, such as apoptosis counteraction, pro-inflammatory cytokines’ release, COX2 expression, NF-kB activation and boosted cellular motility. As cancer may arise when the same regulatory pathways are affected, it is conceivable to hypothesize that CNF1-producing *E. coli* infections can contribute to cancer development. This review focuses on those aspects of CNF1 related to transformation, with the aim of contributing to the identification of a new possible carcinogenic agent from the microbial world.

## 1. Introduction

At the end of the nineteenth century, with the discovery that bacteria can cause some of the major diseases, the idea that bacterial infections might lead to cancer was born even if this assumption is definitively accepted only in a few cases. The most known example is *Helicobacter pylori*, which, while establishing chronic infections in the stomach, is associated with an increased risk of gastric adenocarcinoma and mucosa associated lymphoid tissue (MALT) lymphoma. Several other bacteria have been indicated as possible contributors to the onset of tumour and its progression, but the actual challenge today is to understand the exact mechanisms by which this occurs.

It is now well established that a huge number of bacteria colonizes our body and, in the last years, human beings have been reconsidered as “superorganisms” in co-evolution with their own indigenous microbial community [1,2]. The vast majority of these microbes (10–100 trillion) inhabits our gastrointestinal tract, and constitutes the human intestinal microbiota [3]. A large number of studies link the intestinal microbiota with a possible risk of colorectal cancer, depending on the microbiota composition [4]. Although the intestinal microbiota is largely beneficial, changes in bacterial populations or in the products of bacterial metabolism may contribute to disease. Recently, differences in the colon microbiota in individuals with colon cancer *versus* those with a normal colonoscopy [5] have been reported. In this context, the analyses revealed significant elevation of the Bacteroides/Prevotella population in cancer patients that appeared to be linked with elevated interleukine-17 (IL-17) producing cells in the mucosa [5]. These results are in line with data showing that intestinal inflammation arises from abnormal immune response to bacterial flora in the intestine of genetically susceptible individuals [6]. The onset of chronic inflammation, which is often a common feature of persistent infections [7,8], is closely related to the carcinogenic process since its products lead to a set of dramatic effects including direct DNA damage, inhibition of apoptosis, stimulation of proliferation or inhibition of cell cycle progression, increased angiogenesis and immunosuppression [9,10,11,12,13]. 

Pathogenic bacteria express a broad range of proteins that interact with host cells and that can directly manipulate the inflammatory reaction, contributing to specific stages in cancer development. These proteins, which include the exotoxins and the effectors that are delivered by bacteria directly into the cytoplasm, perturb cellular processes such as proliferation, apoptosis and differentiation, all of which are intimately associated with carcinogenesis. Similarly, their ability to promote anchorage-independent growth could facilitate metastatic potential and lead to cancer progression.

One of the major inhabitants of the intestine is represented by *Escherichia coli*. Although belonging to the normal human intestinal flora, this bacterium becomes highly pathogenic following the acquisition of genes coding for virulence factors, enabling these strains to avoid host defences, colonize extraintestinal sites, and cause tissue damage and disease [14]. These virulence factors are represented by different molecules, including the toxin cytotoxic necrotizing factor type 1 (CNF1), whose gene (*cnf1*) was found in some cases of cancer-associated *E. coli* [15].

## 2. The CNF1 Protein: Structure and Activity

CNF1-producing *E. coli* have occasionally been detected in isolates from faeces of children with diarrhoea, but, more frequently, are responsible for extraintestinal infections, such as septicemia, neonatal meningitis and particularly, urinary tract infections [16]. Also, they have been isolated from soft tissue infections [17]. First described in 1983 by Caprioli and coworkers as a toxin capable of causing multinucleation (“cytotoxic”) in cultured cells and necrosis in rabbit skin (“necrotizing”) [18,19], CNF1 is a single chain multidomain protein toxin of 113.8 kDa with three distinct domains [20]: a *N*-terminal binding domain that interacts with the host cell [21], a *C*-terminal enzymatic moiety, which modifies a specific cellular target in the host cell cytosol and a central domain involved in the toxin translocation into the cytoplasm [22,23].

CNF1 binds with high affinity the surface of epithelial cells via the laminin receptor [24], although it remains to be established if the toxin binds with more affinity to the mature form of this receptor, or its 37 kDa precursor. It has also been demonstrated that CNF1 cell binding is mediated by HSPGs (heparan sulfate proteoglycans). Indeed, treatment of HeLa cells with sodium chlorate, which blocks the synthesis of HSPGs, retarded the uptake of CNF1 [25]. After binding, the toxin is internalized by both clathrin-dependent or independent endocytosis pathways, and is subsequently transferred to an endosomal compartment by a microtubule dependent mechanism [26]. At this level, conformational changes resulting from the acidification of late endosomes drive the translocation of the enzymatic domain into the cytoplasm [23] where CNF1 is cleaved in an approximately 55-kDa fragment that is necessary for full biological activity of the toxin [27]. 

**Figure 1:**
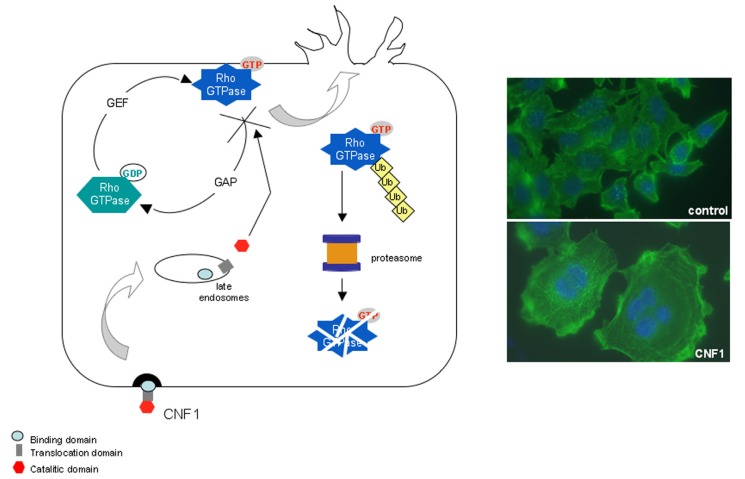
Mechanism of action of CNF1. CNF1 is a single chain multidomain protein toxin that contains a binding domain at the *N*-terminus, a central translocation domain, and an enzymatic domain at *C*-terminus. CNF1 exerts its deamidating activity on a glutamine residue located in the switch 2 domain of the Rho GTPases, essential for the molecule inactivation by GTP hydrolysis. CNF1, by modifying glutamine into glutamic acid, stabilizes the G proteins in their GTP-bound active form enabling them to exert a permanent activity on their effectors. By activating these GTPase, CNF1 stimulates the actin cytoskeleton, fostering a prominent ruffling activity. The activated Rho GTPases are subsequently recognized for ubiquitylation and degraded in the proteasome.

The cytoplasmatic target of CNF1 is represented by the small G proteins belonging to the Rho family, important molecular switches that cycle between a GTP-bound active state and a GDP-bound inactive state, under the strict control of activators (guanine nucleotide exchange factors, GEFs) and inactivators (GTPase-activating proteins, GAPs) [28], to regulate many cellular processes through the binding to downstream effectors. The enzymatic activity of CNF1 consists in the deamidation of a specific glutamine residue (glutamine 63 in Rho [29,30] and glutamine 61 in Rac and Cdc42 [31]) located in the switch 2 domain of the Rho GTPase and essential for the molecule inactivation by GTP hydrolysis [32]. Therefore, by modifying glutamine into glutamic acid, CNF1 stabilizes the G proteins in their GTP-bound active form enabling them to exert a permanent activity on their effectors. The threshold of activation of Rho proteins by CNF1 is, however, attenuated because of a concomitant decrease of their cellular levels, due to the depletion of activated Rho GTPases by the ubiquitin-mediated proteasomal degradation [33]. To be addressed to proteasome, proteins must be first ubiquitynilated through a complex molecular mechanism that involves a cascade of transfer reactions between ubiquitin-carrier proteins [34]. In this process, the ubiquitin-ligase E3 plays a pivotal role, conferring substrate specificity to the reaction [34,35]. In CNF1-treated cells, it was found that the inhibitors of apoptosis proteins (IAPs) [36], which possess a RING domain with E3 ubiquitin ligase activity as well as the tumour suppressor HACE1 [37]—a HECT-domain containing E3 ubiquitin ligase—play an important role for degradation of Rac1. In particular, HACE1-null MEF cells expressed increased levels of Rac1 and a faster migration than control cells [38]. Moreover, Smurf, another HECT domain-containing protein, is responsible for the ubiquitylation of activated RhoA, since CNF1-induced ubiquitylation and proteasome destruction of activated RhoA is impaired in *Smurf1*^−/−^ cells [39].

A schematic representation of the mechanism of action of CNF1 is shown in Figure 1.

## 3. CNF1 Provokes Rho GTPase-Dependent Cellular Effects

The best characterized function of the Rho GTPases is the control of actin cytoskeleton dynamics and organization, which in turn governs a broad range of cellular aspects, including shape, adhesion, motility, cellular junctions remodelling and phagocytosis. CNF1 is widely recognized as an actin polymerization inducer [40,41,42] and this ability brings about the acquisition of new skills by the cells. In fact, it has been demonstrated that CNF1 stimulates, in cultured epithelial cells, macropinocytosis, a Rho-dependent phagocytic-like behaviour [42,43] that is, possibly, the route of entry for CNF1-producing *E. coli.* However, this phenomenon is more complex as it seems plausible that it is the subsequent Rho GTPases’ ubiquitylation and degradation that allows pathogenic bacteria to enter more easily into the host cell and to enhance their pathogenicity. In fact, it has recently been demonstrated the need of Rac1 for the internalization of bacteria into cells triggered by CNF1 together with the recruitment—by ubiquitylated Rac1—of the ubiquitin-binding proteins Tollip and the Tollip-binding proteins, Tom1 and clathrin [44]. Moreover, the activity of CNF1, with its ability to switch on the Rho GTPases by their degradation in the proteasome, is somehow similar to the activity of the intracellular bacterium Salmonella [45], for which a link with cancer has been evidenced [46]. This bacterium first activates the Rho GTPases, by the GEF-like toxin SopE, to promote macropinocytosis that allows its entry into cells and soon after, once inside, deactivates the GTPases via a GAP-mimicking protein (SptP), thus allowing a moderate threshold of Rho protein activation for a high invasion efficiency [47].

The modulation of the actin cytoskeleton via the CNF1-activated Rho GTPases may play a crucial role in certain aspects of the malignant phenotype. In particular, CNF1 induces (i) tumour cell motility caused by cell junctions disruption in uroepithelial 804G cells [33]; (ii) invasiveness, and metastasis [33,48]; (iii) impairment of cytokinesis, thus leading to multinucleation; (iv) nuclear segmentation, amitotic cell division, multipolar mitosis and modulation of autophagy [49]. In addition, as described in the section below, CNF1 protects epithelial cells from apoptosis [50,51], thus probably favouring the survival of cells that have acquired genomic instability. All these cellular phenomena are frequently observed in different types of cancer cells [52,53] and hence, considered as cancer signatures. 

It is worth noting that CNF1 also blocks the G2/M transition in epithelial cells [54] as well as interferes with muscle cell differentiation [55] and can therefore be included in the family of cyclomodulins, bacterial toxins and effectors that interfere with the eukaryotic cell cycle [56,57]. The ability of CNF1 to block cell cycle progression suggests a host strategy that limits damage, whereby specific cellular responses rather than rapid cell death are induced. This could in turn facilitate the bacterial invasion of underlying tissues. The bacteria-engulfing activity of CNF1, linked to its ability to switch on the Rho GTPases, probably enables, not only CNF1-producing *E. coli*, but also other pathogenic microbes to exert their potentially harmful and presumably transforming activity inside the cell, where they can escape the host immune system attack.

## 4. CNF1 Modifies Mitochondrial Architecture and Hinders Apoptosis via the Pro-Inflammatory Akt/IKK/NF-kB Pathway

**Figure 2:**
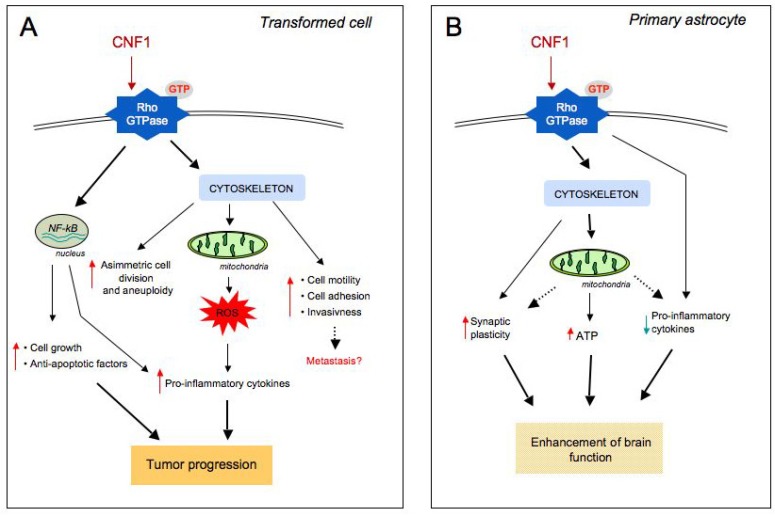
Signalling pathways triggered by CNF1-promoted Rho activation differ depending on the cell type. (**A**) In CNF1-challenged transformed cells, NF-kB translocates from cytoplasm to nucleus where it leads to the expression of pro-inflammatory and anti-apoptotic factors. Modulation of the actin cytoskeleton via the CNF1-activated Rho GTPases also plays a crucial role in certain aspects of the malignant phenotype. In particular, CNF1 induces: tumour cell motility, modification of cellular shape, loss of adhesion with consequent invasiveness and metastasis, an asymmetric cell division and aneuploidy. Furthermore, CNF1 provokes mitochondrial release of reactive oxygen species (ROS) with consequent pro-inflammatory cytokines expression. (**B**) In primary brain cells, through a cytoskeleton modulation, CNF1 acts on mitochondrial activity, boosts cellular ATP content, decreases pro-inflammatory cytokines expression, and increments synaptic plasticity. This leads, *in vivo*, to an enhancement of brain functional performances.

Signalling pathways favouring cell survival can also be considered as pro-transforming factors. We have reported that CNF1 can protect transformed epithelial cells from apoptotic stimuli by (i) overexpressing anti-apoptotic members of the Bcl-2 family [51]; (ii) protecting against the UVB-induced drop of the mitochondrial membrane potential [51] and (iii) activating the pro-inflammatory Rac1/Akt/NF-kB pathway [58]. Rho GTPases are crucially involved in development of inflammatory processes and the Nuclear Factor-kB (NF-kB) represents a well-known key player between Rho, chronic inflammation and cancer [59]. In the context of the CNF1 activity, NF-kB [60] (Figure 2B) is responsible for the ability of the toxin to stimulate the expression of pro-inflammatory factors [61] and to protect host cell from apoptotic stimuli [50,51,58]. As concerns this last point, we have shown that CNF1 also increases the expression of proteins related to cell adhesion (integrins, Focal Adhesion Kinase, cadherins, catenins), thus improving cell spreading and the ability of cells to adhere to each other and to the extracellular matrix [50]. In fact, prolonged cell survival, together with increased adhesion to matrix components might have significant biological consequences and affect the tumorigenic potential of epithelial cells. 

Finally, we have demonstrated that CNF1, by activating the Rac1/Akt/NF-kB pathway, can induce, in epithelial cells, the formation of a complex network of elongated and interconnected mitochondria with an increased average length [58]. Importantly, Bcl-2 silencing reduces the ability of CNF1 to protect cells against apoptosis and also prevents the CNF1-induced mitochondrial changes. Mitochondria are highly dynamic cellular components that undergo continuous cycles of fusion and fission influenced, for instance, by oxidative stress, cellular energy requirements, or the cell cycle state. New important functions beyond energy production have been attributed to mitochondria, such as the regulation of cell survival, because of their role in the modulation of apoptosis, autophagy, and aging [62]. Therefore, since the mitochondrial remodelling is pivotal for the role of these organelles in cell physiology and the mitochondrial dysfunction can contribute to a number of human disorders, including cancer [63], the role of CNF1 as a factor favoring transformation can be further supported by this novel finding.

Furthermore, it is well known that the Rho GTPases can stimulate the production of reactive oxygen species (ROS) [64]. This consequently triggers the activation of NF-kB that in turn controls the expression of genes involved in inflammation, cell growth, and suppression of apoptosis [65,66]. 

In fact, CNF1 causes in transformed cells a Rac-dependent super oxide anion release [67] and enhances ROS-dependent pro-inflammatory cytokines’ production [61]. Furthermore, the infection promoted by this toxin leads to a general inflammatory process, triggering the synthesis and release of proinflammatory cytokines, such as IL-6, IL-8 and TNF-, from uroepithelial [61] and endothelial [68] cells. The pro-inflammatory role of CNF1 is also supported by the ability of the toxin to strongly up-regulate the transcription of cyclooxygenase-2 (COX-2) [69], an immediate-early gene induced in response to pro-inflammatory cytokines, tumor promoters, and growth factors and over-expressed in cancers of the lung, colon, stomach, and breast [70].

On the whole, it appears that CNF1 touches some of the signalling pathways that are engaged by carcinogens and tumor promoters, as schematized in Figure 2A.

## 5. Evidence of a Link between CNF1 and Cancer in Humans

Therefore, considering the so far described activity of CNF1 on cells, the crucial question is if CNF1 can be linked to the onset or development of cancer in humans. Humans are colonized by residential microbes that do not passively inhabit the host. In fact, there is increasing evidence for a rich, complex, dynamic, and individual-specific microbial interaction with the host and human cancers that should now be considered against the background of host-microbiome interactions [4].

In the case of *E. coli*, it has been reported that adherent-invasive *E. coli* are associated specifically with intestinal mucosa of patients with Crohn’s disease while the bacterial association with intestinal mucosa of healthy individuals is low [71]. Another study demonstrated that, when the overlying mucus layer is removed, the normal colonic mucosa is relatively free from aerobic bacteria, whereas Crohn’s disease mucosae, the surface of colon cancers, and the distant mucosae from colon cancer resection specimens contain relatively plentiful aerobic flora, particularly *E. coli* [72]. The study of colonic mucosa-associated *E. coli* from patients with colorectal cancer (CRC) or diverticulosis indicated that *E. coli* strains producing CNF1 colonized colon cancers more frequently than they did diverticulosis samples. Moreover, cyclomodulins encoding genes (especially *cnf1*) were over-represented in colon cancers and the distal colon cancers were more frequently colonized by *E. coli* producing cyclomodulins with respect to diverticulosis samples [15]. 

On the assumption that bacteria are associated with CRC, bacterial strains present at the origin of cancer may disappear and be replaced by other bacteria better adapted to the cancer environment. Consequently, it remains difficult to determine whether the increase in specific bacteria is the consequence of the presence of malignant tissues or the cause of the cancer. However, these toxin-producing bacteria, which colonize the malignant tumours, probably have an impact on the evolution of CRC.

Recent next-generation sequencing studies of the intestinal microbiota now offer an unprecedented view of the aetiology of sporadic CRC and have revealed that the microbiota associated with CRC contains bacterial species that differ in their temporal associations with developing tumours. According to the bacterial driver-passenger model [73], different members of the intestinal microbiota can be involved in the initiation and progression phases of the neoplastic process. In particular, the carcinogenic process can be initiated by driver bacteria and then promoted by passenger bacteria or neoplastic-promoting microorganisms that outcompete drivers in the tumour microenvironment. 

Taking into account the mechanism of action of CNF1 and what is above reported on this toxin, our hypothesis is that CNF1-producing *E. coli* act as passenger bacteria, reinforcing and favouring but not causing the development of colorectal cancer.

## 6. Conclusions: CNF1, a Janus Toxin Playing with Cell Regulation

As stated above, the colonic mucosa of patients at risk for CRC is intrinsically colonized by pathogenic bacteria that can function as “drivers”. These bacteria can cause inflammation, increased cell proliferation and/or the production of genotoxic substances that contribute to the accumulation of mutations during the cellular transformation. This oncogenic process is accompanied by alteration of the microenvironment with a selective pressure on the local microbiota and with the gradual replacement of “driver” bacteria by “passenger” bacteria, this one consisting of tumour-foraging opportunistic pathogens, commensal or probiotic bacteria with a competitive advantage in the tumour niche [73]. Passenger bacteria can force through cancer only those tissues already transformed, but in themselves they cannot induce transformation. This would be the effect of CNF1. 

In fact, the hypothesis of CNF1-producing *E. coli* acting as “passenger” bacteria is intriguing and is in keeping with the lower ability of some cancer epithelial lines, if compared to human primary cells, to ubiquitylate specific Rho proteins. This finding entails the risk to maintain the Rho GTPases permanently activated in cancer cells challenged with CNF1. Furthermore, the above hypothesis is also reinforced by the interesting effect of CNF1 in non transformed brain cells [74] and *in vivo* on the central nervous system of pathological mouse models [75,76]. On primary brain cells, the CNF1 effect is characterized by an increased development of neuritis with a wider dendritic tree and richer synapse content [74]. *In vivo*, a single intracerebroventricular injection of CNF1 is able to modulate Rho GTPases activity and to improve cognitive performances in animal models of Alzheimer’s disease [76] and Rett syndrome [75]. Interestingly, in Alzheimer disease, which is characterized by an enhanced inflammatory status, the toxin is able to counteract high levels of pro-inflammatory cytokines expression, behaving exactly in the opposite way of how it behaves in transformed cells. Thus, it is plausible to hypothesize a more articulated effect of CNF1 that depends on the cell microenvironment, on the cell condition, as well as on the cell types (Figure 2A,B). 

These are the reasons why we have called CNF1 a “janus toxin”. Janus is the two-faced god of beginnings and transitions, and looks to the future and the past. Janus is also the god of doors: one face for doors open one for doors closed. In fact, CNF1 has two faces, one that activates the GTPases and the other one that destroys them. Also, CNF1 apparently behaves as a friend with its powerful therapeutic potential and as a foe for the carcinogenic signatures in its activity. Further *in vitro* and *in vivo* studies are required to establish whether and how CNF1 can represent an “enemy within” as a new possible carcinogenic agent from the microbial world.
